# Measuring cardiomyocyte cellular characteristics in cardiac hypertrophy using diffusion‐weighted MRI

**DOI:** 10.1002/mrm.29775

**Published:** 2023-06-22

**Authors:** Mohsen Farzi, Sam Coveney, Maryam Afzali, Marie‐Christine Zdora, Craig A. Lygate, Christoph Rau, Alejandro F. Frangi, Erica Dall'Armellina, Irvin Teh, Jürgen E. Schneider

**Affiliations:** ^1^ Biomedical Imaging Science Department, Leeds Institute of Cardiovascular and Metabolic Medicine University of Leeds Leeds UK; ^2^ Cardiff University Brain Research Imaging Centre (CUBRIC), School of Psychology Cardiff University Cardiff UK; ^3^ Diamond Light Source Ltd. Harwell Science and Innovation Campus Didcot UK; ^4^ Department of Physics & Astronomy University College London London UK; ^5^ Division of Cardiovascular Medicine, Radcliffe Department of Medicine University of Oxford Oxford UK; ^6^ Centre for Computational Imaging and Simulation Technologies in Biomedicine (CISTIB), School of Computing University of Leeds Leeds UK

**Keywords:** biophysical models, cardiac microstructure mapping, diffusion‐weighted MRI, synchrotron X‐ray imaging

## Abstract

**Purpose:**

This paper presents a hierarchical modeling approach for estimating cardiomyocyte major and minor diameters and intracellular volume fraction (ICV) using diffusion‐weighted MRI (DWI) data in ex vivo mouse hearts.

**Methods:**

DWI data were acquired on two healthy controls and two hearts 3 weeks post transverse aortic constriction (TAC) using a bespoke diffusion scheme with multiple diffusion times (Δ), q‐shells and diffusion encoding directions. Firstly, a bi‐exponential tensor model was fitted separately at each diffusion time to disentangle the dependence on diffusion times from diffusion weightings, that is, b‐values. The slow‐diffusing component was attributed to the restricted diffusion inside cardiomyocytes. ICV was then extrapolated at Δ=0 using linear regression. Secondly, given the secondary and the tertiary diffusion eigenvalue measurements for the slow‐diffusing component obtained at different diffusion times, major and minor diameters were estimated assuming a cylinder model with an elliptical cross‐section (ECS). High‐resolution three‐dimensional synchrotron X‐ray imaging (SRI) data from the same specimen was utilized to evaluate the biophysical parameters.

**Results:**

Estimated parameters using DWI data were (control 1/control 2 vs. TAC 1/TAC 2): major diameter—17.4 μm/18.0 μm versus 19.2 μm/19.0 μm; minor diameter—10.2 μm/9.4 μm versus 12.8 μm/13.4 μm; and ICV—62%/62% versus 68%/47%. These findings were consistent with SRI measurements.

**Conclusion:**

The proposed method allowed for accurate estimation of biophysical parameters suggesting cardiomyocyte diameters as sensitive biomarkers of hypertrophy in the heart.

## 1 INTRODUCTION

Cardiac microstructure plays a fundamental role in the electro‐mechanical function of the heart, and its dysregulation is a key determinant of heart failure.^1^, ^2^ Despite this importance, characterization of cellular‐level biophysical parameters remains challenging. Biophysical models of diffusion‐weighted MRI (DWI) data are a promising way for quantifying biomarkers of cardiac microstructure including cell size, volume fraction, and dispersion.^3^ Unlike signal representation techniques such as diffusion tensor imaging that yield an indirect characterization of tissue‐specific properties,^4^, ^5^ biophysical models aim to directly quantify cellular features by simplifying the underlying tissue environment as a combination of basic geometrical compartments.^3^, ^6^

Reports on biophysical models of cardiac diffusion MRI have been very limited so far. Hsu et al.^7^ proposed a bi‐exponential tensor model to measure intracellular volume fraction (ICV). To measure cell radius, Kim et al.^8^ proposed a two‐compartment model where intra‐ and extracellular space were represented by impermeable cylinders and unrestricted isotropic tensors, respectively. In a more comprehensive study, Farzi et al.^3^ examined a range of two‐compartment models in the heart where cardiomyocytes were represented by four different cylinder models including a standard cylinder, cylinder with an elliptical cross‐section (ECS), cylinders with Gamma distributed radii, and cylinders with Bingham distributed axes, respectively. The extracellular space was represented by an isotropic tensor (ball) or an oblate tensor (pancake).

Despite the effort to develop and validate cardiac biophysical models,^3^, ^7^, ^8^ unresolved challenges still exist. First, dependence on diffusion time has solely been attributed to the intracellular space using a variation of an impermeable cylinder. Diffusion of water molecules in the extracellular space could also be highly restricted due to the small interstitial gaps between myocardial sheetlets. Ignoring this time dependency could either result in underestimated intracellular volume fraction or overestimated cell radius. This issue has also been observed in axonal diameter mapping in the brain.^6^ Second, acquisition artifacts, noise propagation, and model inadequacy lead to errors in the estimated parameters. The magnitude of these errors in relation to the intrinsic biological variation is only incompletely understood. Measuring the accuracy of estimated biophysical parameters is an essential consideration for their adoption in practice. Poor parameter estimation accuracy could adversely affect their sensitivity to cardiac pathologies and limit their usefulness. Third, a reliable validation strategy based on a three‐dimensional (3D) reconstruction of individual cardiomyocytes has not yet been established.

This paper aims to develop and validate a new biophysical modeling technique to quantify ICV and cardiomyocyte diameters in healthy and diseased myocardium ex vivo and assess the sensitivity of the estimated biophysical parameters to cardiac disease. To disentangle the diffusion time dependence on intra‐ and extracellular diffusivities from diffusion weightings, a two‐step fitting procedure is proposed here. In the first step, a bi‐exponential tensor model is fitted to DWI data collected at different encoding directions and b‐values, but with similar diffusion times. Collecting the volume fraction for the slow‐diffusing component at multiple diffusion times, the decrease in ICV in relation to the increase in diffusion time is modeled using linear regression, and ICV is reported at diffusion time equals zero. In the second step, assuming a cylinder‐ECS model for cardiomyocytes, the major and minor diameters were estimated by fitting the model parameters to the secondary and tertiary diffusion eigenvalues for the slow‐diffusing component. The accuracy of estimated model parameters was assessed experimentally against the ground‐truth biophysical parameters quantified from the corresponding Synchrotron Radiation Imaging (SRI) scans from the same specimen, and numerically using simulations in the presence of noise. Building on our previously established SRI‐based 3D virtual histology for quantification of ICV and cardiomyocytes' orientation,^3^, ^9^ we further extend the framework to quantify cardiomyocyte diameters automatically for validation. Finally, a murine disease model of transverse aortic constriction (TAC) was used to evaluate the performance of the biophysical model under a wider range of (patho‐) physiological conditions. TAC creates a pressure overload that is comparable to aortic stenosis in humans, resulting in well‐characterized hypertrophy as cardiomyocytes compensate by adding contractile units and thereby increasing cellular cross‐sectional area.^10^

## 2 THEORY

The total normalized signal S is modeled as a linear combination of signals from two nonexchanging compartments attributed to the intracellular (IC) and the extracellular (EC) space.^3^, ^7^, ^8^ The restricted diffusion inside cardiomyocytes is modeled using a cylinder with an ECS ($S_{\text{cyl‐ECS}}$) whereas the extracellular space and the vascular components are lumped into one effective compartment represented by a tensor ($S_{\text{tensor}}$) similar to Farzi et al.^3^

(1) $$S=v_{\text{IC}}S_{\text{cyl‐ECS}}+v_{\text{EC}}S_{\text{tensor}},$$

where $\left\{v_{\text{IC}},v_{\text{EC}}\}\in [0,1\right]$ are volume fractions for each compartment, respectively. By construction, $v_{\text{IC}}+v_{\text{EC}}=1$.

The normalized signal for a cylinderECS model^3^ is

(2) $$\begin{array}{ll} S_{\text{cyl‐ECS}} & =\exp[-|g{|}^{2}L_{\|}(d;\Delta ,\delta ){\left({\hat{g}}^{T}{\hat{u}}_{1}\right)}^{2}]\\ & \;\exp[-|g{|}^{2}L_{⊥}(r_{1},d;\Delta ,\delta ){\left({\hat{g}}^{T}{\hat{u}}_{2}\right)}^{2}]\\ & \;\exp[-|g{|}^{2}L_{⊥}(r_{2},d;\Delta ,\delta ){\left({\hat{g}}^{T}{\hat{u}}_{3}\right)}^{2}], \end{array}$$

where |g| and $\hat{g}$ are the gradient magnitude and direction, Δ is the diffusion time, δ is the diffusion gradient duration, d is the parallel diffusivity along the cylinder axis ${\hat{u}}_{1}$, and $r_{1}$ and $r_{2}$ are the major and minor radii along ${\hat{u}}_{2}$ and ${\hat{u}}_{3}$, respectively. The functions $L_{\|}$ and $L_{⊥}$ are defined as follows^11^:

(3) $$L_{\|}(d;\Delta ,\delta )=\gamma^{2}\delta^{2}(\Delta -\delta /3)d,$$

(4) $$\begin{array}{llllll} L_{⊥}(r,d;\Delta ,\delta )=2\gamma^{2}\overset{\infty }{\underset{m=1}{\sum }} & {\left[d^{2}\beta_{m}^{6}(r^{2}\beta_{m}^{2}-1)\right]}^{-1}\; & \ldots & \; & & \\ & \left[2d\beta_{m}^{2}\delta -2+\right.\; & \ldots & \; & & \\ & 2\exp[-d\beta_{m}^{2}\delta ]+ & \ldots & \; & & \\ & 2\exp[-d\beta_{m}^{2}\Delta ]-\; & \ldots & \; & & \\ & \exp[-d\beta_{m}^{2}(\Delta -\delta )]-\; & \ldots & \; & & \\ & \left.\exp[-d\beta_{m}^{2}(\Delta +\delta )]\right].\; & & \; & & \end{array}$$

Here, $\beta_{m}$ is the mth root of equation $J_{1}^{\prime }(\beta_{m}r)=0$ and $J_{1}^{\prime }$ is the derivative of the Bessel function of the first kind, order one. The normalized signal for a tensor model with symmetric diffusion tensor D and diffusion weighting factor $b=\gamma^{2}\delta^{2}(\Delta -\delta /3)|g{|}^{2}$ is:

(5) $$\begin{array}{ll} S_{\text{tensor}} & =\exp[-b{\hat{g}}^{T}\text{D}\hat{g}]=\\ & \;\exp[-bd_{\|}{\left({\hat{g}}^{T}{\hat{u}}_{1}\right)}^{2}]\\ & \;\exp[-bd_{{⊥}_{1}}{\left({\hat{g}}^{T}{\hat{u}}_{2}\right)}^{2}]\\ & \;\exp[-bd_{{⊥}_{2}}{\left({\hat{g}}^{T}{\hat{u}}_{3}\right)}^{2}], \end{array}$$

where $d_{\|}$, $d_{{⊥}_{1}}$, and $d_{{⊥}_{2}}$ are primary, secondary, and tertiary diffusion eigenvalues, respectively, for tensor D and ${\hat{u}}_{1}$, ${\hat{u}}_{2}$, and ${\hat{u}}_{3}$ are the corresponding diffusion eigenvectors. Diffusion eigenvectors are assumed to be parallel with the corresponding axes from the cylinderECS model.

To disentangle the DWI signal dependence on diffusion time and diffusion weighting (b‐values), we propose a two‐step hierarchical fitting procedure.

### 2.1 Step 1

To account for signal dependence on diffusion weightings, diffusion time is fixed at this step. By setting the diffusion time Δ at a fixed value, the cylinderECS model can be represented by a standard diffusion tensor D where (cf. Equations 2 and 5):

(6) $$\begin{array}{ll} d_{\|} & =\frac{L_{\|}(d;\Delta ,\delta )}{\gamma^{2}\delta^{2}(\Delta -\delta /3)}=d, \end{array}$$

(7) $$\begin{array}{ll} d_{{⊥}_{i}} & =\frac{L_{⊥}(r_{i},d;\Delta ,\delta )}{\gamma^{2}\delta^{2}(\Delta -\delta /3)}\;\text{for}i=1,2. \end{array}$$

By replacing the cylinerECS model with a standard diffusion tensor model, Equation (1) can be rewritten as a tensor‐tensor model:

(8) $$S=v_{\text{IC}}S_{\text{tensor}}+v_{\text{EC}}S_{\text{tensor}},$$

with the parameter vector

(9) $$p=\left[v_{\text{IC}},d_{\text{IC}}^{\|},d_{\text{IC}}^{{⊥}_{1}},d_{\text{IC}}^{{⊥}_{2}},d_{\text{EC}}^{\|},d_{\text{EC}}^{{⊥}_{1}},d_{\text{EC}}^{{⊥}_{2}},\theta ,\phi ,\alpha \right],$$

where θ, ϕ, and α are rotation angles to represent diffusion eigenvectors; $\theta =\arccos({\hat{u}}_{1}^{T}\hat{z})$ is the angle between the z‐axis and ${\hat{u}}_{1}$, $\phi =\arctan(\frac{{\hat{u}}_{1}^{T}\hat{y}}{{\hat{u}}_{1}^{T}\hat{x}})$ is the angle between the x‐axis and the projection of ${\hat{u}}_{1}$ on the xy‐plane, and $\alpha =\arctan(\frac{{\hat{u}}_{3}^{T}\hat{z}}{-{\hat{u}}_{2}^{T}\hat{z}})$ is the angle between the ${\hat{u}}_{1}$ rotated by $90^{\circ }$ about the z‐axis and ${\hat{u}}_{2}$.^3^

Given an observed data vector $\tilde{s}$ with M measurements, the parameter vector p for a tensor‐tensor model is estimated at each diffusion time separately by maximizing the log‐likelihood $\ell (p,\sigma |\tilde{s})\equiv \log 𝒫(\tilde{s}|p,\sigma )$ where σ is the SD of noise. At high signal‐to‐noise ratio (SNR), a Gaussian distribution can approximate the noise statistics;

(10) $$\ell (p,\sigma |\tilde{s})=-\frac{1}{2\sigma^{2}}\overset{M}{\underset{m=1}{\sum }}{\left[S(p;\Psi_{m})-{\tilde{s}}_{m}\right]}^{2}-\frac{M}{2}\ln2\pi \sigma^{2},$$

where Ψ is the set of all imaging parameters, and $S(p;\Psi_{m})$ is the analytic synthesized signal from the compartment model. For a normally distributed noise, minimizing the negative log‐likelihood is equivalent to minimizing the root mean squared error between the observed data and the predicted analytic signal from the model.

Given a $v_{\text{IC}}$ measurement obtained at each diffusion time, the ICV is estimated using a linear regression model to extrapolate its value as Δ→0.

### 2.2 Step 2

Given $d_{\text{IC}}^{{⊥}_{1}}$ and $d_{\text{IC}}^{{⊥}_{2}}$ measurements obtained at each diffusion time, $r_{1}$ and $r_{2}$ were estimated by minimizing the cost function below:

(11) $$𝒥=\underset{\Delta^{\prime }=10,\ldots ,50\;ms}{\sum }{\left[d_{\text{IC}}^{{⊥}_{i}}(\Delta^{\prime })-\frac{L_{⊥}(r_{i},d^{\|};\Delta^{\prime },\delta )}{\gamma^{2}\delta^{2}(\Delta^{\prime }-\delta /3)}\right]}^{2},$$

where $d^{\|}$ is estimated as Δ→0 by fitting $d^{\|}(\Delta )=d(\Delta =0)+c_{1}\Delta$ to intracellular parallel diffusivities estimated at different diffusion times from step one.

In Step 1, the primary diffusion eigenvalue in the extracellular space was set at 2.1 $\mu m^{2}$/ms corresponding to the free diffusivity in the buffer measured at an region‐of‐interest (ROI) in the left ventricular cavity. All experiments were conducted using a Matlab toolkit publicly available at https://github.com/mfarzi/myoscope.

### 2.3 Experimental sampling requirements

To reliably measure model parameters in Step 1, multishell and multidirection data are essential. Bi‐exponential signal decay can only be observed for large b‐values exceeding 1500 s/${\text{mm}}^{2}$. In Step 2, to accurately measure cardiomyocyte diameters, experiments with multiple diffusion times are necessary. Specifically, diameters in the range of 20 μm can only be estimated with diffusion times longer than 30 ms.

## 3 METHODS

### 3.1 In vivo MRI

All experimental investigations conformed to the UK Home Office guidance on the Operations of Animals (Scientific Procedures) Act 1986 incorporating European Directive 2010/63/EU and were approved by the University of Oxford Animal Welfare and Ethical Review Board. TAC surgery and in vivo MRI occurred under isoflurane general anesthesia, with buprenorphine hydrochloride (1 mg/kg) provided for peri‐ and post‐surgical analgesia. Mice were euthanized via overdose of pentobarbitone and rapid excision of the heart.

In vivo cine imaging was performed on healthy female controls and male mice 3 weeks post TAC (C57Bl/6J, *n* = 2 each) as described previously.^12^, ^13^ In brief, a double‐gated, two‐fold compressed‐sensing accelerated multiframe gradient echo cine images (echo time/pulse repetition time =1.79/4.6 ms, field of view =30×30 mm, slice thickness 1 mm, matrix‐size 128×128), covering the entire left ventricle (LV) were acquired in short‐axis orientation, using a 9.4T preclinical MR scanner (Agilent) with shielded gradients (max gradient strength = 1 T/m, rise time = 130 μs) and a quadrature‐driven birdcage coil (Rapid Biomedical) of inner diameter = 33 mm. End‐systolic and end‐diastolic frames were manually segmented using a bespoke software tool to quantify global LV function and to measure end‐diastolic wall thickness in a mid‐ventricular slice.

### 3.2 Heart samples

Sample preparation was performed as described previously^14^: the hearts were excised and perfused in constant pressure Langendorff mode at 80 mmHg with modified Krebs‐Henseleit solution and cardioplegically arrested with STH‐2 buffer. The hearts were then perfused via an aortic cannula at constant flow with 4% paraformaldehyde (PFA) and subsequently with 1% PFA. The hearts were immersed in 1% PFA and stored at $4^{\circ }$C to continue fixation. Prior to imaging, the hearts were rinsed of fixative via immersion in phosphate‐buffered saline (PBS) and perfusion of PBS by aortic cannula. The hearts were then embedded in 2% agarose‐PBS gel (Web Scientific) to minimize sample motion for MRI and subsequent synchrotron imaging.

### 3.3 DWI data acquisition and postprocessing

DWI was performed on the same preclinical MR scanner but with a quadrature‐driven birdcage coil of inner diameter = 20 mm (Rapid Biomedical). Images were acquired using a DW fast spin echo sequence with six gradient strengths, five diffusion times (Δ = 10, 20, 30, 40, and 50 ms), and 10 diffusion‐encoding directions similar to Farzi et al.^3^ One non‐DW image was also acquired for each diffusion time, bringing the number of images to 305. The imaging parameters were: 3D fast spin echo sequences with bandwidth = 100 kHz, resolution = 187.5μm isotropic, field‐of‐view = 9×9×5 mm, echo train length = 8, echo spacing = 3.4 ms, diffusion gradient duration δ = 2.5 ms, and nominal b‐value = 69, 280, 620, 1100, 1700, and $2500\;s/{\text{mm}}^{2}$. For b‐value = $2500\;s/{\text{mm}}^{2}$, the gradient strengths were 780.06, 530.98, 430.76, 370.76, and 330.70 mT/m for diffusion times 10, 20, 30, 40, and 50 ms, respectively. The gradient strengths were adjusted to achieve lower b‐values. Fully sampled data with a single average were acquired. No parallel imaging technique was used. The total acquisition time for MR imaging was 37 h for each mouse heart. To improve the SNR, dynamic receiver gain adjustment was used,^14^ and a low‐pass Butterworth filter of order n=4 with a normalized cutoff frequency of 1/3 was applied.^3^ Similar to Kim et al.,^8^ different echo time were used for each diffusion time. To compensate for this variable echo time, diffusion signals were normalized to the corresponding $s_{0}$, that is, signal measured at b=0, for each diffusion time.

### 3.4 SRI data acquisition and postprocessing

**FIGURE 1:**
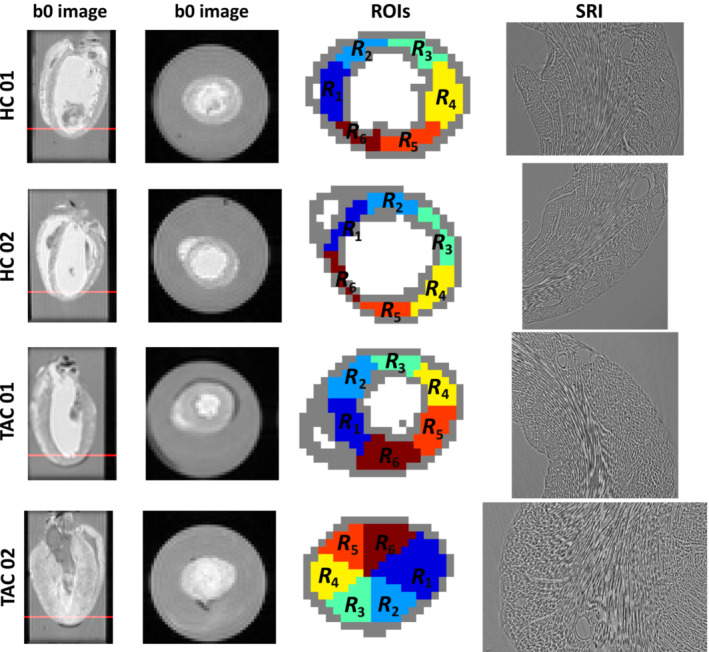
Ex vivo diffusion‐weighted MRI (DW‐MRI) and corresponding synchrotron X‐ray imaging (SRI) slices. The first column shows the ex vivo DWI measurements at *b* = 0 (no diffusion weighting) in the four‐chamber view for the four mouse hearts. The second column shows the cross‐section in the short‐axis orientation at *b* = 0 (no diffusion weighting) corresponding to the red line in the four‐chamber view. The third column shows the corresponding DWI mask where the left mid‐ventricle wall is segmented into six region‐of‐interests (ROIs): $R_{1}$ in the septum, $R_{2}$ and $R_{3}$ in the anterior wall, $R_{4}$ in the lateral wall, and $R_{5}$ and $R_{6}$ in the inferior wall. The boundary voxels shown in gray were excluded from the quantification to avoid the partial volume effect. The last column shows reconstructed SRI images from collected tomographic data corresponding to $R_{4}$ in the short‐axis orientation. Lighter intensities represent the extracellular space whereas the darker intensities represent cardiomyocytes.

Tomographic SRI data were acquired at beamline I13‐2 imaging branch of the Diamond Light Source as previously described.^3^, ^9^, ^15^ In brief, a single ROI near the apex was imaged using monochromatic X‐rays (20–30 keV). The exposure time per projection image was ≈1.2 s and 2401 projection scans were acquired at uniform angular spacing over 180 degrees of sample rotation. The total acquisition time was 1 h 25 min. The 2D projections were combined using a filtered back‐projection algorithm^16^ to obtain a 3D volume with an effective isotropic pixel size of 2.2μm as detailed in our previous work.^3^ Next, reconstructed SRI scans were rigidly registered to DWI data using the tensor‐based warping method proposed by Farzi et al.^3^ such that structure tensors (STs)^17^, ^18^ were aligned with the diffusion tensors. Figure 1 shows one cross‐section of the reconstructed SRI data in the short‐axis orientation registered with the corresponding DWI data for two healthy control (HC) hearts and two disease TAC hearts.

#### 3.4.1 SRI Data Quantification

**FIGURE 2:**
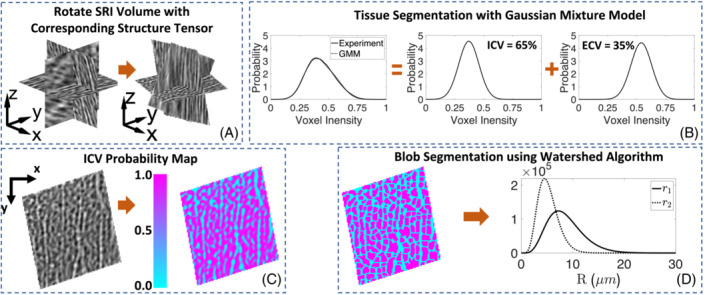
Proposed synchrotron X‐ray imaging (SRI) Quantification Pipeline. Each diffusion‐weighted MRI (DWI) voxel corresponds to a volume of 85×85×85 voxels in the reconstructed SRI scans. (A) First, the SRI volume is rotated to align cardiomyocytes with the z‐axis. This rotation is essential to estimate cardiomyocytes' cross‐sections appropriately in the xy‐plane. The rotation matrix was derived by first computing the structure tensors (STs) using the method of quadrature filters,^18^ and then estimating ST eigenvectors and combining them. (B) The gray‐level intensity profile for the intra‐ and extracellular space was assumed to be Gaussian and a Gaussian Mixture Model (GMM) was fitted to the data. The lighter intensities represent the extracellular space whereas the darker intensities represent the cardiomyocytes. (C) Given the GMM model, a probability map for how likely each pixel belongs to the intracellular space is computed in the xy‐plane. (D) To segment cardiomyocytes' cross‐sections, a threshold of 0.5 is applied to each voxel. Next, at each xy‐plane, cardiomyocyte cross‐sections were segmented as individual blobs using a watershed algorithm,^20^ and the major and minor radii were estimated for each blob. A Gamma distribution is then fitted to the estimated radii over all blobs in the SRI volume corresponding to a single DWI voxel.

Figure 2 shows a schematic framework to quantitate ICV and cardiomyocytes' minor and major radii from the reconstructed SRI scans. In this study, each DWI voxel corresponds to a volume of 85×85×85 voxels in the reconstructed SRI scans. First, structure tensors (STs)^17^, ^18^ were computed based on gray level intensity gradients in the reconstructed SRI scans using the method of *quadrature filters* performed in the spatial domain using freely available Matlab codes.^19^ Similar to Farzi et al.,^3^ quadrature filtering was performed using a spatial filter size of 11 with center frequency π/3 and bandwidth of 2 octaves. Next, each voxel is rotated so the tertiary ST eigenvector corresponding to the primary diffusion eigenvector is aligned with the z‐axis. This is essential to segment cardiomyocytes' cross‐sections perpendicular to their longitudinal axis properly. An intensity‐based clustering approach was then used to segment cardiomyocytes. Since the intensity profile shows one dominant mode in its histogram, differentiating between intra‐ and extracellular space is challenging. Here, like Farzi et al.,^3^ the gray‐level intensity profile for each cluster was assumed to be Gaussian. A Gaussian Mixture Model (GMM) is then fitted to data estimating a probability map for how likely each pixel belongs to the intracellular space. ICV is then estimated as the mean of the probability map.

To segment cardiomyocytes' cross‐sections, each voxel is assigned to the most likely cluster, that is, a threshold of 0.5 is applied to the estimated intracellular probability map. Next, cardiomyocyte cross‐sections were segmented as individual blobs at each xy−plane using a watershed algorithm.^20^ The major and minor radii were estimated for each blob. A Gamma distribution is then fitted to the estimated radii over all blobs in the SRI volume, corresponding to a single DWI voxel.

### 3.5 Degeneracy and Precision Analysis

A global minimum at $p^{*}$ in the likelihood function means that $\ell (p^{*}|s)\leq \ell (p|s)$ for all parameters p. The optimization problem is *degenerate* if at least two different biophysically plausible sets of model parameters $p_{1}$ and $p_{2}$ exist such that $\ell (p_{1}|s)=\ell (p_{2}|s)=\ell (p^{*}|s)$ for the measurement data s. To assess the degeneracy of the objective cost function ℓ(p|s) for a single draw of noise and one specific set of parameters, each biophysical parameter $p_{i}$ was swept within its physiologically plausible range, and the minimum cost function was computed by optimizing the remaining parameters.^21^, ^22^, ^23^ These graphs (see Figure S3) provide a useful one‐dimensional representation to assess if multiple local or global minima exist for a specific data vector s. If one global minimum exists in all graphs, then a unique solution exists, and the objective function is nondegenerate. The global minimum in these graphs is the same as the optimal solution for ℓ(p|s). Furthermore, degeneracy may not be a property of the model alone, but also a consequence of the choice of acquisition parameters.

For a parameter vector p and the corresponding noisy data vector s generated from the model, the difference between the estimated parameter $\hat{p}$ and the ground‐truth p, that is, $\hat{p}-p$, is the error of the maximum likelihood estimator (MLE) for $\hat{p}$. An ensemble of $\hat{p}-p$, known as the *sampling distribution* of the MLE, can be generated using different draws of random noise for the same p and obtaining the MLE estimate for each noise draw. The estimator is *unbiased* if the mean of the estimator error is zero. The precision of the estimator can also be measured by calculating the SD of the estimation error. A smaller SD means the MLE of a parameter varies less around its mean value. Notably, under certain conditions, the variance of the sampling distribution can be related to the Fisher Information.

To assess the accuracy of parameter estimation for a tensor‐tensor model, synthetic data was simulated using $p=\left[v_{\text{IC}},d_{\text{IC}}^{\|},d_{\text{IC}}^{{⊥}_{1}},d_{\text{IC}}^{{⊥}_{2}},d_{\text{EC}}^{\|},d_{\text{EC}}^{{⊥}_{1}},d_{\text{EC}}^{{⊥}_{2}},\theta ,\phi ,\alpha \right]$ $=\left[0.6,0.9,0.5,0.3,2.1,1.6,1.0,0,0,0\right]$ with diffusivities and rotation angles reported in $\mu m^{2}/\text{ms}$ and radian, respectively. Intracellular eigenvectors were parallel to the extracellular eigenvectors. DWI data was generated using the same diffusion scheme used to collect the experimental data in this study, that is, 10 diffusion directions and six nonzero b‐values= 69, 280, 620, 1100, 1700, and 2500 $\text{ms}/\mu m^{2}$. One b0 measurement was also simulated. Next, *N* = 1000 noisy samples at SNR = 20, 30, 40, 60, 80, and 100 dB were generated using a Rician noise model.^24^ All SNR were computed with respect to the b0 image. At each SNR level, the model parameters were estimated for each noisy sample of measurements. The mean and SD of the estimator error $\hat{p}-p$ were reported.

### 3.6 Dependence on diffusion Time

Kim et al.^8^ showed that the diffusion of water molecules inside the cardiomyocytes are restricted by their size. This experiment identified whether signal dependence on diffusion times also exists in the extracellular space. To assess the dependence of diffusion eigenvalues on diffusion time, a tensor‐tensor model was fitted at Δ=10,20,30,40, and 50 ms separately using six different *b*‐values and 10 diffusion encoding directions. An ROI comprised of 37 voxels was selected inside the LV cavity filled with the buffer. We expect free diffusion of water molecules in the buffer with no diffusion time dependence. The mid‐ventricle wall was segmented into six ROIs: R1 in the septum, R2 and R3 in the anterior wall, R4 in the lateral wall, and R5 and R6 in the inferior wall (Figure 1). The average and SD for both intra‐ and extracellular diffusion eigenvalues were reported.

## 4 RESULTS

### 4.1 In vivo MRI

Mid‐ventricular end‐diastolic frames in short axis orientation are shown in Figure S1 for all four hearts. Ejection fraction was substantially reduced in the TAC hearts (35% and 28% vs. 50% in controls), while left ventricular mass was increased (TAC: 122/156 mg vs. 61/96 mg in controls) (Table 1). The difference was less pronounced when normalized to the body weight (TAC: 4.7/6.0 $\times 10^{-3}$ vs. 4.5/3.4 $\times 10^{-3}$ in controls). Mid‐left‐ventricular end‐diastolic wall thickness was 1.2/1.1 mm in the TAC and 0.94/0.85 mm in the control hearts, suggesting a hypertrophic response in the TAC mice as expected.

**TABLE 1:** Analysis of cardiac morphology and function using in vivo MRI.

Sample	EDV (μL)	ESV (μL)	SV (μL)	EF (%)	LVM (mg)	BW (g)	EDWTH (mm)
HC01	73.1	36.5	36.6	50.1	95.5	21.4	0.94
HC02	45.7	23.4	22.3	48.8	61.5	18.2	0.85
TAC01	73.3	47.3	26	35.5	122.1	25.8	1.08
TAC02	99.1	71.7	27.5	27.7	156.1	26.1	1.23

Abbreviations: BW, body weight; EDV, end diastolic volume; EDWTH, mean end diastolic wall thickness; EF, ejection fraction; ESV, end systolic volume; LVM, left ventricle mass; SV, stroke volume.

### 4.2 Degeneracy analysis

**FIGURE 3:**
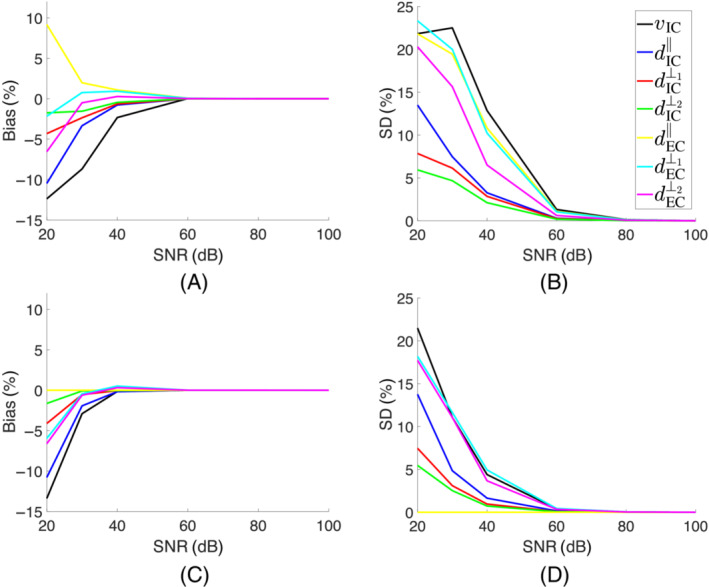
Precision analysis for the Tensor‐Tensor model. Panels (A) and (B) show the mean and SD for the maximum likelihood estimator error. Panels (C) and (D) show the mean and SD of the estimator error when $d_{\text{EC}}^{\|}$ is a priori fixed at 2.1$\mu m^{2}/\text{ms}$. The reported figures were normalized to 1 for ICV and 3.0$\mu m^{2}/\text{ms}$ for the diffusivities. For SNR≥40 dB and fixed $d_{\text{EC}}^{\|}$, the bias and variance were below 1% and 5%, respectively. Synthetic data was simulated using model parameters $p=\left[v_{\text{IC}},d_{\text{IC}}^{\|},d_{\text{IC}}^{{⊥}_{1}},d_{\text{IC}}^{{⊥}_{2}},d_{\text{EC}}^{\|},d_{\text{EC}}^{{⊥}_{1}},d_{\text{EC}}^{{⊥}_{2}},\theta ,\phi ,\alpha \right]$ $=\left[0.6,0.9,0.5,0.3,2.1,1.6,1.0,0,0,0\right]$ with diffusivities and rotation angles reported in $\mu m^{2}/\text{ms}$ and radian, respectively. DWI data was generated using ten diffusion directions and six nonzero *b*‐values = 69, 280, 620, 1100, 1700, and 2500 $\text{ms}/\mu m^{2}$. One b0 measurement was also simulated bringing the total number of measurements to 10×6+1=61. Next, N=1000 noisy samples were generated at SNR = 20, 30, 40, 60, 80, and 100 dB using a Rician distribution.

For the simulated signal s using a tensor‐tensor model with $p=\left[v_{\text{IC}},d_{\text{IC}}^{\|},d_{\text{IC}}^{{⊥}_{1}},d_{\text{IC}}^{{⊥}_{2}},d_{\text{EC}}^{\|},d_{\text{EC}}^{{⊥}_{1}},d_{\text{EC}}^{{⊥}_{2}},\theta ,\phi ,\alpha \right]=\left[0.6,0.9,\right.\left.0.5,0.3,2.1,1.6,1.0,0,0,0\right]$, a unique global minimum exists for the objective cost function, confirming that the cost function is nondegenerate (Figure S3). As the SNR tends to infinity, the estimator variance approaches zero, allowing parameter estimation with high accuracy (Figure 3). However, as the SNR decreases, both the estimator variance and bias increase, and parameter estimation becomes challenging. Figure S2 shows the distribution of estimated $v_{\text{IC}}$, $d_{\text{IC}}^{\|}$, and $d_{\text{EC}}^{\|}$ at a high SNR = 100 dB and a lower practical SNR = 40 dB, for example.

As a practical solution to improve the estimator precision, extracellular primary diffusion eigenvalue $d_{\text{EC}}^{\|}$ was fixed to 2.1 $\mu m^{2}/\text{ms}$ in this study. Figure 3D shows the SD of estimator error in this scenario. Enforcing this constraint reduces the normalized SD for all parameters to below 5% at SNR ≥ 40 dB.

### 4.3 Dependence on diffusion time

**FIGURE 4:**
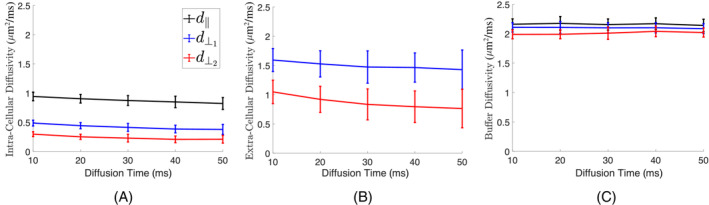
Dependence of intra‐ and extracellular diffusion eigenvalues on diffusion time. (A) The primary, secondary, and tertiary diffusion eigenvalues decreased by 13%, 22%, and 29% for the slow‐diffusing compartment by increasing the diffusion time from 10 to 50 ms. (B) The secondary and tertiary diffusion eigenvalues decreased by 10% and 27% by increasing the diffusion time from 10 to 50 ms, respectively. Data is shown on the mid‐ventricle wall for the first control heart. (C) No time dependency was observed in the buffer diffusivity measured in an ROI selected inside the left ventricular cavity. The mean for the diffusion eigenvalues in the buffer was 2.1 $\mu m^{2}/\text{ms}$. The vertical error bar below and above each data point indicates 1 SD.

The mean diffusivity inside the buffer was measured as 2.1 $\mu m^{2}/\text{ms}$. No diffusion time dependence was seen in the buffer (Figure 4C). Both intra‐ and extracellular apparent diffusivities depend on diffusion times (Figure 4). For the control hearts, the primary, secondary, and tertiary eigenvalues decreased by about 10%, 20%, and 30% for the slow‐diffusing compartment. The secondary and tertiary diffusion eigenvalues decreased by about 10% and 30%, respectively. Similar patterns were also seen for the TAC heart, but the secondary diffusion eigenvalue in the extracellular space showed a slightly higher reduction by 6%. Figure 4 shows data for the first control heart.

### 4.4 Biophysical parameters

Tables 2, 3, 4 show the mean and SD for ICV, major diameter, and minor diameter in six ROIs for both control and TAC hearts (Figure 1). The average ICV was 62% in the control hearts. ICV estimations were similar in both control hearts across the six ROIs (Table 2). Unlike the controls, ICV was different between the TAC hearts: ICV = 68% and 47% in the first and second TAC hearts, respectively. Estimated ICV was homogeneous in all six ROIs in the second TAC heart, and about 20% lower than the controls. Estimated ICV in the first TAC heart were similar to controls in regions $R_{2}$, $R_{3}$, and $R_{4}$, whereas a slight increase of about 5% was seen in regions $R_{1}$, $R_{5}$, and $R_{6}$. The major and minor cardiomyocyte diameters were homogeneous in all six ROIs in the controls. The cardiomyocyte cross‐section in SRI data suggested an ECS with $r_{1}/r_{2}\approx 2$ on average. The major and minor cardiomyocyte radii were both consistently higher by about 30% in the TAC heart compared to the controls in all six ROIs within the LV wall (Tables 3 and 4). The reported increase in cardiomyocyte diameters from the ground‐truth SRI measurement was in accordance with the estimated parameters from the compartment models. However, the SD for estimated parameters was higher using compartment modeling compared to the ground‐truth SRI quantification.

**TABLE 2:** Average (SD) of intracellular volume fraction estimated in six regions‐of‐interest (%).

Sample	Data	$R_{1}$	$R_{2}$	$R_{3}$	$R_{4}$	$R_{5}$	$R_{6}$	$\cup R_{i}$
	SRI	64 ± 0.7	61 ± 1.2	56 ± 2.3	55 ± 1.4	62 ± 2.4	65 ± 0.9	60 ± 4.6
HC01	DWI 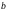	50 ± 7.4	45 ± 4.0	44 ± 4.9	46 ± 9.6	46 ± 6.3	44 ± 5.8	46 ± 7.4
	DWI 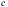	65 ± 7.1	57 ± 6.4	57 ± 7.5	65 ± 8.6	62 ± 7.6	61 ± 7.5	62 ± 8.1
	SRI	64 ± 3.1	65 ± 2.7	66 ± 1.4	63 ± 6.7	63 ± 2.0	64 ± 1.5	64 ± 3.9
HC02	DWI 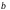	42 ± 6.2	44 ± 6.8	51 ± 4.9	45 ± 4.4	49 ± 1.9	48 ± 5.0	46 ± 5.9
	DWI 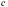	56 ± 3.8	59 ± 8.0	67 ± 4.4	62 ± 5.3	64 ± 3.8	63 ± 3.8	62 ± 6.3
	SRI	74 ± 2.0	69 ± 2.8	62 ± 1.3	67 ± 2.4	72 ± 1.4	74 ± 3.2	71 ± 4.4
TAC01	DWI 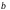	56 ± 5.9	53 ± 4.7	51 ± 3.3	51 ± 4.8	55 ± 4.4	57 ± 3.9	54 ± 5.0
	DWI 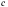	70 ± 4.0	66 ± 6.7	65 ± 5.1	67 ± 5.3	70 ± 6.4	69 ± 4.9	68 ± 5.8
	SRI	43 ± 2.1	42 ± 2.7	45 ± 2.1	45 ± 2.1	44 ± 1.8	41 ± 2.0	43 ± 2.6
TAC02	DWI 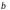	35 ± 5.9	33 ± 5.9	40 ± 4.8	40 ± 6.4	35 ± 6.3	32 ± 5.0	36 ± 6.4
	DWI 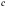	50 ± 7.2	45 ± 6.2	48 ± 6.3	48 ± 8.7	44 ± 6.3	46 ± 6.4	47 ± 7.3

*Note*: The grey shading represents the ground‐truth SRI measurements for each sample heart.

Abbreviations: HC, healthy control; TAC, transverse aortic constriction.

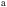
 See Figure 1 for a visual demonstration of the six selected ROIs $R_{1}$ to $R_{6}$. To avoid the partial volume effect, averaging is performed over voxels in the mid‐ventricle wall only.

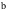
The cylinderECS‐tensor model with the one‐step fitting procedure.

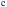
The cylinderECS‐tensor model with the proposed two‐step hierarchical fitting procedure.

**TABLE 3:** Average (SD) of minor cardiomyocyte diameter estimated in six regions‐of‐interest (ROIs) (μm).

Sample	Data	$R_{1}$	$R_{2}$	$R_{3}$	$R_{4}$	$R_{5}$	$R_{6}$	$\cup R_{i}$
	SRI	10.8 ± 0.4	10.6 ± 0.2	9.6 ± 0.4	9.4 ± 0.2	10.6 ± 0.6	11.2 ± 0.4	10.2 ± 0.8
HC01	DWI 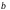	12.2 ± 5.6	10.2 ± 3.4	9.8 ± 2.4	10.0 ± 6.6	9.6 ± 2.4	9.6 ± 2.8	10.4 ± 4.8
	DWI 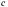	10.2 ± 1.4	10.8 ± 1.2	10.8 ± 1.6	9.8 ± 1.4	10.4 ± 1.2	10.4 ± 1.2	10.2 ± 1.4
	SRI	10.2 ± 0.2	10.2 ± 0.2	10.0 ± 0.1	10.2 ± 0.4	9.8 ± 0.1	10.0 ± 0.2	10.0 ± 0.2
HC02	DWI 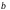	8.6 ± 2.4	8.0 ± 2.6	11.0 ± 3.2	7.2 ± 3.0	8.8 ± 1.2	11.2 ± 2.0	8.8 ± 2.8
	DWI 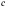	9.6 ± 1.2	8.8 ± 1.4	10.2 ± 0.8	9.0 ± 1.0	9.8 ± 0.8	10.4 ± 0.6	9.4 ± 1.2
	SRI	13.6 ± 0.4	13.0 ± 0.6	12.4 ± 0.6	13.0 ± 1.0	13.6 ± 1.0	13.8 ± 0.6	13.4 ± 0.8
TAC01	DWI 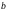	13.2 ± 2.0	15.0 ± 3.2	15.0 ± 3	13.2 ± 2.2	13.6 ± 2.4	14.2 ± 1.8	14.0 ± 2.4
	DWI 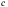	12.0 ± 1.2	13.4 ± 0.8	13.8 ± 1.8	12.4 ± 1.0	12.8 ± 1.6	12.8 ± 1.0	12.8 ± 1.4
	SRI	11.4 ± 0.4	11.8 ± 0.8	12.0 ± 0.4	12.2 ± 0.4	11.8 ± 0.6	11.2 ± 0.4	11.8 ± 0.6
TAC02	DWI 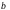	12.0 ± 3.6	15.8 ± 4.6	13.0 ± 2.4	13.6 ± 2.0	12.4 ± 2.2	12.8 ± 3.2	13.2 ± 3.4
	DWI 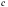	13.2 ± 1.6	14.2 ± 1.8	13.2 ± 1.8	13.8 ± 1.0	13.0 ± 1.4	13.2 ± 2.0	13.4 ± 1.8

*Note*: The grey shading represents the ground‐truth SRI measurements for each sample heart.

Abbreviations: HC, healthy control; TAC, transverse aortic constriction.

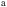
 See Figure 1 for a visual demonstration of the six selected ROIs $R_{1}$ to $R_{6}$. To avoid the partial volume effect, averaging is performed over voxels in the mid‐ventricle wall only.

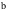
The cylinderECS‐tensor model with the one‐step fitting procedure.

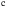
The cylinderECS‐tensor model with the proposed two‐step hierarchical fitting procedure.

**TABLE 4:** Average (SD) of major cardiomyocyte diameter estimated in six regions‐of‐interests (ROIs) (μm).

Sample	Data	$R_{1}$	$R_{2}$	$R_{3}$	$R_{4}$	$R_{5}$	$R_{6}$	$\cup R_{i}$
	SRI	17.6 ± 0.8	17.2 ± 0.4	16.0 ± 0.6	15.8 ± 0.6	16.8 ± 0.6	18.0 ± 0.6	16.8 ± 1.0
HC01	DWI 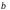	19.2 ± 3.4	19.4 ± 4.4	21.6 ± 6.8	19.0 ± 5.2	17.0 ± 3.0	18.2 ± 2.2	19.0 ± 4.6
	DWI 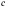	17.6 ± 2.0	17.2 ± 2.0	19.0 ± 2.8	17.0 ± 1.8	16.8 ± 2.2	17.2 ± 1.8	17.4 ± 2.2
	SRI	16.6 ± 0.4	16.4 ± 0.2	16.2 ± 0.2	16.4 ± 0.8	15.8 ± 0.2	16.2 ± 0.6	16.2 ± 0.6
HC02	DWI 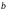	20.6 ± 3.6	21.4 ± 4.4	19.0 ± 1.8	20.6 ± 2.8	17.8 ± 1.6	20.2 ± 3.8	20.0 ± 3.4
	DWI 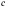	18.6 ± 2.4	18.8 ± 2.8	17.6 ± 0.8	18.4 ± 1.2	16.6 ± 1.2	17.8 ± 3.0	18.0 ± 2.0
	SRI	21.0 ± 0.6	20.2 ± 0.8	19.8 ± 0.4	20.6 ± 1.2	21.0 ± 1.6	21.4 ± 0.8	20.8 ± 1.0
TAC01	DWI 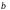	22.8 ± 3.6	19.8 ± 3.6	22.2 ± 2.8	19.4 ± 3.6	18.2 ± 2.2	25.2 ± 7.2	21.4 ± 5.2
	DWI 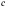	20.0 ± 2.8	18.0 ± 2.2	19.4 ± 2.4	17.2 ± 2.8	16.6 ± 1.8	23.0 ± 7.4	19.2 ± 4.6
	SRI	21.6 ± 0.8	22.0 ± 1.0	22.2 ± 0.6	22.2 ± 0.4	21.8 ± 0.6	21.4 ± 0.8	21.8 ± 0.8
TAC02	DWI 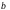	19.8 ± 4.6	23.4 ± 7.2	22.0 ± 4.0	18.8 ± 2.6	20.0 ± 3.8	23.4 ± 6.0	21.0 ± 5.2
	DWI 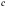	18.4 ± 2.6	20.2 ± 5.4	19.8 ± 2.8	18.0 ± 1.8	17.8 ± 1.8	19.8 ± 2.6	19.0 ± 3.2

*Note*: The grey shading represents the ground‐truth SRI measurements for each sample heart.

Abbreviations: HC, healthy control; TAC, transverse aortic constriction.

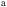
 See Figure 1 for a visual demonstration of the six selected ROIs $R_{1}$ to $R_{6}$. To avoid the partial volume effect, averaging is performed over voxels in the mid‐ventricle wall only.

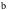
The cylinderECS‐tensor model with the one‐step fitting procedure.

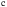
The cylinderECS‐tensor model with the proposed two‐step hierarchical fitting procedure.

**FIGURE 5:**
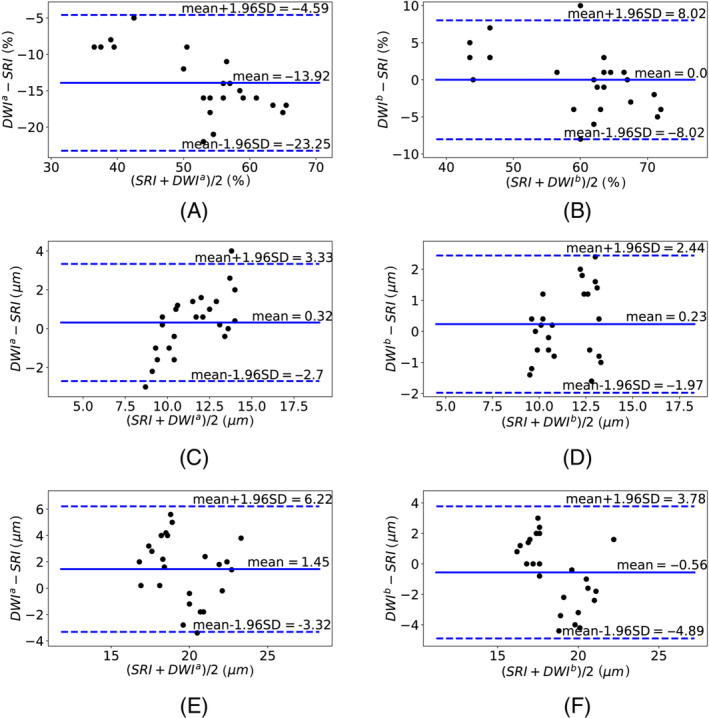
Bland–Altman analysis between synchrotron X‐ray imaging (SRI) and diffusion‐weighted MRI (DWI) parameters. The Bland–Altman plots show the average difference between the measurements on the y‐axis and the mean of the two measurements on the x‐axis. A small bias (the solid line) would confirm consistency between measurements. To assess the agreement between the two measurements, dashed lines represent the interval range of values within which 95% of the differences between the two methods are expected to lie. The column on the left compares SRI and DWI data modeling using a one‐step optimization, whereas the column on the right shows similar results for comparison between SRI and DWI data modeling using the proposed two‐step optimization method. The rows show results for ICV, minor, and major diameters from top to bottom, respectively. (A,B) ICV; (C,D) minor diameter; (E,F) major diameter.

Figure 5 shows the Bland–Altman analysis to assess the agreement between SRI and DWI data in biophysical parameter measurement. Biophysical parameters estimated from DWI data using the proposed two‐step hierarchical optimization approach were consistent with SRI measurements. However, DWI data modeling using one‐step optimization resulted in a large ICV underestimation of about 15%.

## 5 DISCUSSION

The study presents four contributions to cardiac microstructure mapping. Firstly, a new hierarchical modeling strategy is proposed to reliably estimate cardiomyocyte diameters and volume fraction. Our findings confirmed that a mono‐exponential signal decay cannot appropriately represent the DWI signal at high b‐values. A bi‐exponential tensor model improved the root mean squared error by approximately 40% compared to diffusion tensor imaging, but cannot account for the signal dependence on diffusion times. A cylinderECS‐tensor model represented the restricted diffusion inside cardiomyocytes well, but resulted in large ICV underestimation (≈15%) by ignoring diffusion time dependence in the extracellular space, as confirmed in this study (Table 2). To alleviate the ICV underestimation issue, previous studies used spherical or planar tensors to model the extracellular space.^3^, ^8^ However, adopting an isotropic tensor modestly improved the ICV estimation error to about 8%. The proposed two‐step hierarchical fitting procedure addressed this issue by disentangling the DWI signal dependence on diffusion times and *b*‐values.

The biexponential tensor model showed that intracellular diffusion eigenvalues depend on diffusion times. For the slow‐diffusing compartment, the primary, secondary, and tertiary eigenvalues decreased by approximately 10%, 20%, and 30% as the diffusion time increased from 10 to 50 ms. The reduction in secondary and tertiary diffusion eigenvalues can be attributed to restricted diffusion inside cardiomyocytes. The modest reduction in the primary diffusion eigenvalue may be attributed to the slight variation in cardiomyocyte orientation within each voxel.^3^ ICV was also modestly dependent on diffusion time decreasing by 5% when increasing the diffusion time from 10 to 50 ms. This reduction in ICV may be due to increased water exchange between compartments at higher diffusion times. For the fast‐diffusing compartment, the tertiary diffusion eigenvalue reduced by about 30% when increasing the diffusion times from 10 to 50 ms, which may be linked to the sheetlet gaps. A modest reduction of 10% was also observed in the second diffusion eigenvalue. This reduction may also be linked with slight dispersion of cardiomyocyte orientation within each voxel.

As the second contribution, we examined the effect of hypertrophy on estimated biophysical parameters in two TAC hearts. At the macroscopic level, the TAC model resulted in thicker LV walls compared to control hearts (Table 1). At the cellular level, this increase in cardiac mass could be attributed to the 30% increase in the cardiomyocyte minor diameter compared to the control hearts (Table 3). SRI data validated these findings, suggesting cardiomyocyte diameters as potential biomarkers of hypertrophy in the heart. The first TAC heart showed a modest increase in ICV while the second TAC heart had a significantly lower ICV compared to the controls. The slight increase of 5% in the first TAC heart is in keeping with the suggestion that an increase in ICV putatively occurs before the onset of irreversible myocardial fibrosis (i.e., decrease in ICV).^25^ The reduction in ICV seen in the second TAC heart could be due to the heart being arrested in a contracted state (cf. Figure 1). ICV depends on cardiomyocyte volume and packing density inside sheetlets as well as the gaps between sheetlets. During heart contraction, shortening of cardiomyocytes or increasing cleavage gaps between sheetlets result in lower ICV.^26^

As the third contribution, we extended our 3D virtual histology framework to quantify cardiomyocyte diameters using an automatic watershed‐based segmentation algorithm and morphological operations. The estimated ICV was consistent with results reported in Greiner et al.^27^ The estimated diameter from reconstructed SRI scans was 30% lower than the value reported in Chen et al.^28^ but consistent with results reported in Farzi et al.^3^ This variation could be attributed to anatomical variation between species.^29^ Our findings showed good consistency between estimated biophysical parameters and the ground‐truth parameters estimated from SRI data (Figure 5). Variation in estimated SRI parameters may be attributed to the physiological or pathophysiological variation, segmentation accuracy, data acquisition artifacts, and measurement noise. However, we hypothesize that the primary source of variation in estimated SRI parameters is physiological. This hypothesis is in keeping with the higher variance observed in estimated biophysical parameters using DWI data. This higher variance may be attributed to the acquisition noise propagation in DWI data.

The fourth contribution is the quantification of the uncertainties in parameter estimation for a biexponential tensor model using simulated data. For the simulated signal s using a tensor‐tensor model with $p=\left[v_{\text{IC}},d_{\text{IC}}^{\|},d_{\text{IC}}^{{⊥}_{1}},d_{\text{IC}}^{{⊥}_{2}},d_{\text{EC}}^{\|},d_{\text{EC}}^{{⊥}_{1}},d_{\text{EC}}^{{⊥}_{2}},\theta ,\phi ,\alpha \right]=\left[0.6,0.9,0.5,\right.\left.0.3,2.1,1.6,1.0,0,0,0\right]$, our findings suggest that a tensor‐tensor model is not degenerate under the proposed diffusion scheme (Figure S3). However, as SNR decreases below  60 dB, the estimated parameters suffer from large bias (>5%) and poor precision (>5%) as shown in Figure 3. As a practical solution, similar to the NODDI model,^30^ we fixed the primary extracellular diffusion eigenvalue at 2.1$\mu m^{2}/\text{ms}$. This parameter fixation allowed for a more accurate estimation of the remaining parameters at realistic experimental SNR levels of  40 dB (Figure 3C,D). Here we used an experimental approach based on synthetic simulations to assess the degeneracy. Employing an analytic approach similar to Coelho et al.^31^ could further help to optimize the number of diffusion directions, shells, and diffusion times to improve the precision of estimated biophysical parameters without fixing the extracellular diffusivities.

This study had the following limitations. The proposed hierarchical fitting procedure represented the diffusion time dependence in the extracellular space indirectly using a separate tensor model per diffusion time. A new compartment model may be required to account for the hindered time‐dependent diffusion signal in the extracellular space in future studies. Similar to References 3, 7, and 8, the vascular component was combined with the interstitial space between cardiomyocytes into one effective compartment in this study. We previously showed that the restriction effects imposed by vessel boundaries would be negligible at diffusion times below 50 ms in this fixed ex vivo experimental setting.^3^ However, a separate vascular compartment will be required to model cardiac perfusion.

To optimize SNR, different echo times were used for each diffusion time. To account for different T2 weighting, collected signals were normalized to their corresponding b0 signal at each diffusion time. However, if the underlying compartments have different T2 values, the relative contributions of the compartments to the signal will not be the same at all echo times due to the difference in the amount of T2 decay. Kim et al.^8^ investigated this effect on a cylinder‐ball model and reported minor effects on estimated biophysical parameters. Therefore, we adopted this approach in our study.

In this study, hearts were fixed using PFA. Fixatives like PFA stabilize the tissue microstructures and make them metabolically inactive, but they could affect the estimated biophysical parameters.^32^ To reduce these effects, hearts were rinsed of excess fixative via immersion in PBS in this study.^33^ Lohr et al.^34^ studied the effects of continuous formalin fixation on diffusion tensor properties; mean diffusivity and fractional anisotropy were reduced by 22% and 10% postfixation after 7 days.^34^

We finally recognize the small sample size as a further limitation. However, this work was designed as a proof‐of‐concept study aimed to establish tools and techniques. Future work will focus on a systematic characterization of the myocardium in health and disease using biophysical modeling. The application to genetically modified mouse models may also help to further elucidate molecular mechanisms driving microstructural alterations.

## 6 CONCLUSIONS

We propose a new biophysical modeling approach to quantify cardiac microstructure in healthy and TAC mouse hearts ex vivo using a biexponential tensor model, followed by a cylinderECS model. We show that both the intra‐ and extracellular diffusion eigenvalues depend on the diffusion time. At realistic SNR levels of approximately 40 dB, a large MLE error, rather than degeneracy, resulted in poor precision in the estimation of biophysical parameters. As a practical solution, we fixed the primary extracellular diffusivity in this study at the measured free diffusivity in the buffer. The estimated ICV and cardiomyocyte radii were consistent with the ground‐truth SRI quantification. The cardiomyocyte minor diameter was a sensitive biomarker of hypertrophy in the heart, demonstrating approximately 30% higher values in the TAC hearts compared to the controls.

## Supporting information




**Figure S1.** In vivo MRI. Representative mid‐ventricular, end‐diastolic cine images in short‐axis orientation for all hearts used in this study. The TAC hearts seem enlarged with increased wall thickness.**Figure S2.** Precision analysis for the tensor‐tensor model. The scatter plots show the distribution of estimated parameters $v_{\text{IC}}$, $d_{\text{IC}}^{\|}$, and $d_{\text{EC}}^{\|}$ versus each other. The top row shows the results at SNR = 100 dB. Estimate parameters were highly concentrated about the ground truth marked by red lines. The bottom row shows the results at SNR = 40 dB. Estimated parameters were highly scattered about the ground‐truth parameters. These results are based on N=1000 data vectors simulated using the same model parameters but with different noise samples drawn from a Rician distribution. See Figure 3 for more information about the data simulation.**Figure S3.** Degeneracy analysis for the Tensor‐Tensor model. At each noise level, one synthetic signal vector was randomly drawn from a Rician noise distribution. All signal vectors were generated using the same model parameters $p=\left[v_{\text{IC}},d_{\text{IC}}^{\|},d_{\text{IC}}^{{⊥}_{1}},d_{\text{IC}}^{{⊥}_{2}},d_{\text{EC}}^{\|},d_{\text{EC}}^{{⊥}_{1}},d_{\text{EC}}^{{⊥}_{2}},\theta ,\phi ,\alpha \right]$ $=\left[0.6,0.9,0.5,0.3,2.1,1.6,1.0,0,0,0\right]$ with diffusivities and rotation angles reported in $\mu m^{2}/\text{ms}$ and radian, respectively. For each parameter, its value was fixed at a specific value within its physiologically plausible range. The remaining parameters were optimised to fit the model to the given input data. The root mean squared error (RMSE) was then reported for each parameter. A global minimum in each graph confirms that a unique solution exists for each optimization problem.
