# Supplementary material for: Measuring cardiomyocyte cellular characteristics in cardiac hypertrophy using diffusion‐weighted MRI
Source: Magn Reson Med. 2023 Jun 22;90(5):2144–57. doi: 10.1002/mrm.29775 (PMC10962572; doi:10.1002/mrm.29775)
Supplement: Supplementary file 1 — Figure S1. In vivo MRI. Representative mid‐ventricular, end‐diastolic cine images in short‐axis orientation for all hearts used in this study. The TAC hearts seem enlarged with increased wall thickness.Figure S2. Precision analysis for the tensor‐tensor model. The scatter plots show the distribution of estimated parameters vIC, dIC‖, and dEC‖ versus each other. The top row shows the results at SNR = 100 dB. Estimate parameters were highly concentrated about the ground truth marked by red lines. The bottom row shows the results at SNR = 40 dB. Estimated parameters were highly scattered about the ground‐truth parameters. These results are based on N=1000 data vectors simulated using the same model parameters but with different noise samples drawn from a Rician distribution. See Figure 3 for more information about the data simulation.Figure S3. Degeneracy analysis for the Tensor‐Tensor model. At each noise level, one synthetic signal vector was randomly drawn from a Rician noise distribution. All signal vectors were generated using the same model parameters p=vIC,dIC‖,dIC⊥1,dIC⊥2,dEC‖,dEC⊥1,dEC⊥2,θ,ϕ,α =0.6,0.9,0.5,0.3,2.1,1.6,1.0,0,0,0 with diffusivities and rotation angles reported in μm2/ms and radian, respectively. For each parameter, its value was fixed at a specific value within its physiologically plausible range. The remaining parameters were optimised to fit the model to the given input data. The root mean squared error (RMSE) was then reported for each parameter. A global minimum in each graph confirms that a unique solution exists for each optimization problem. [file MRM-90-2144-s001.pdf]

Measuring Cardiomyocyte Cellular Characteristics in Cardiac Hypertrophy using Diffusion-Weighted MRI

Mohsen Farzi[1], Sam Coveney [1], Maryam Afzali[1,2], Marie-Christine Zdora[4,5], Craig A. Lygate[3], Christoph Rau[4], Alejandro F. Frangi[6], Erica Dall'Armellina[1], Irvin Teh[1], Jürgen E. Schneider[1,*]

1 Biomedical Imaging Science Department, Leeds Institute of Cardiovascular and Metabolic Medicine, University of Leeds, Leeds LS2 9JT, UK.

2 Cardiff University Brain Research Imaging Centre (CUBRIC), School of Psychology, Cardiff University, Cardiff CF24 4HQ, UK.

3 Division of Cardiovascular Medicine, Radcliffe Department of Medicine, University of Oxford, Oxford OX3 9DU, UK.

4 Diamond Light Source Ltd, Didcot, Oxfordshire OX11 0DE, UK.

5 Department of Physics & Astronomy, University College London, London WC1E 6BT, UK.

6 Centre for Computational Imaging and Simulation Technologies in Biomedicine (CISTIB), School of Computing, University of Leeds, Leeds LS2 9JT, UK.

Supplementary Figures

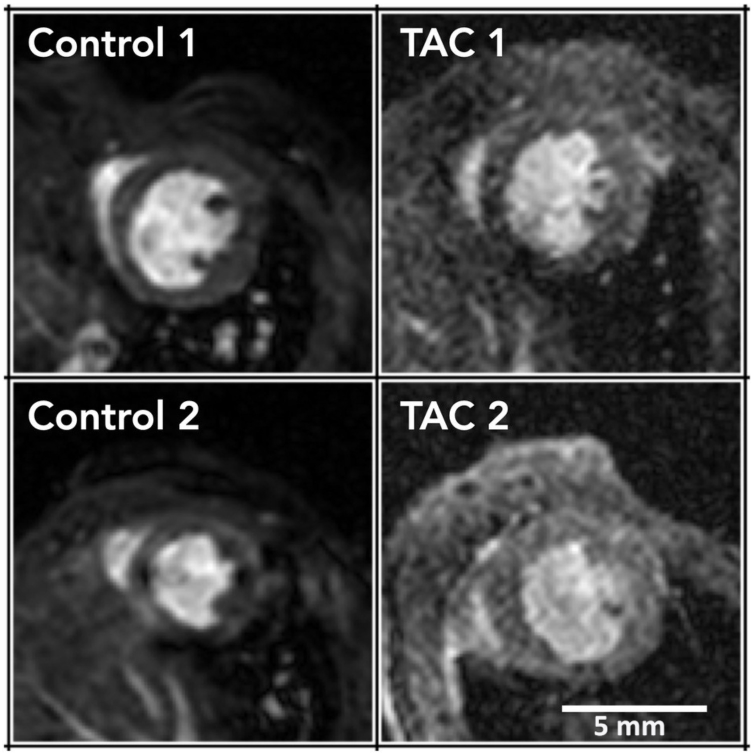

Figure S1: **In Vivo MRI**

Representative mid-ventricular, end-diastolic cine images in short-axis orientation for all hearts used in this study. The TAC hearts seem enlarged with increased wall thickness.

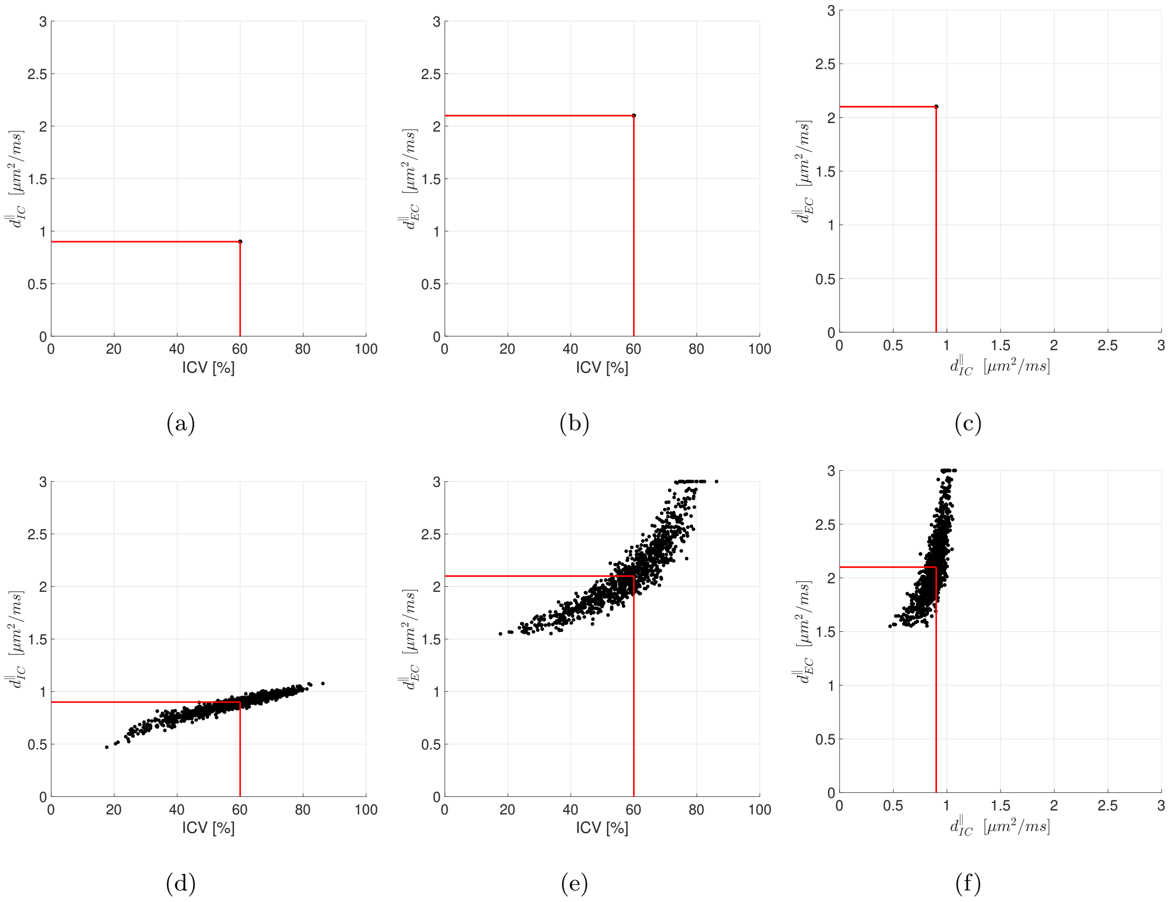

Figure S2: **Precision analysis for the tensor-tensor model**

The scatter plots show the distribution of estimated parameters v_{IC}, $d^{\|}_{\mathrm{IC}}$, and $d^{\|}_{\mathrm{EC}}$ versus each other. The top row shows the results at SNR=100 dB. Estimate parameters were highly concentrated about the ground truth marked by red lines. The bottom row shows the results at SNR=40 dB. Estimated parameters were highly scattered about the ground-truth parameters. These results are based on $N = 1000$ data vectors simulated using the same model parameters but with different noise samples drawn from a Rician distribution. See Figure 3 for more information about the data simulation.

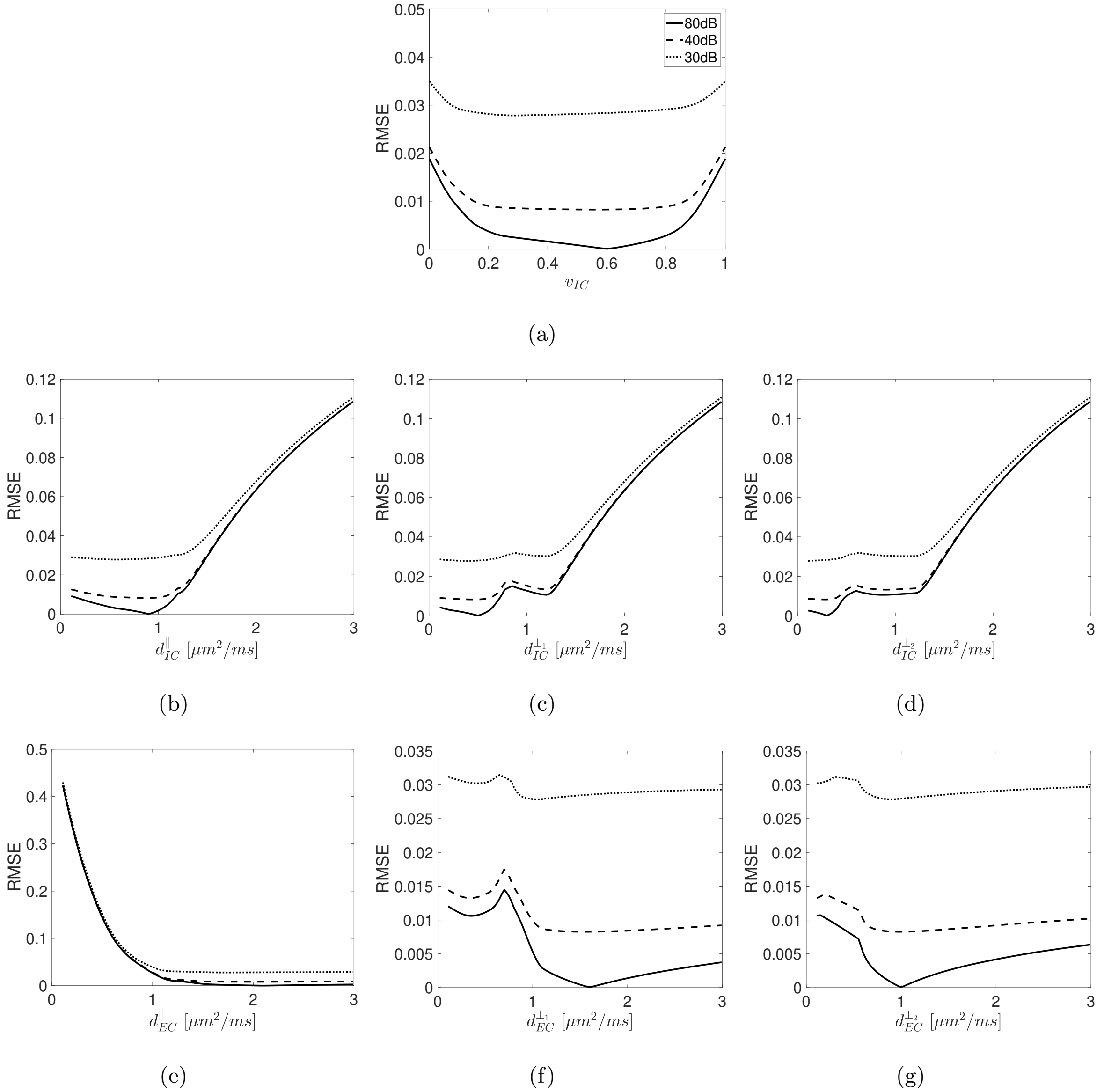

Figure S3: **Degeneracy analysis for the Tensor-Tensor model.**

At each noise level, one synthetic signal vector was randomly drawn from a Rician noise distribution. All signal vectors were generated using the same model parameters $\boldsymbol{p} = \left[v_{\mathrm{IC}}, d_{\mathrm{IC}}^{\parallel}, d_{\mathrm{IC}}^{\perp_1}, d_{\mathrm{IC}}^{\perp_2}, d_{\mathrm{EC}}^{\parallel}, d_{\mathrm{EC}}^{\perp_1}, d_{\mathrm{EC}}^{\perp_2}, \theta, \phi, \alpha\right] = [0.6, 0.9, 0.5, 0.3, 2.1, 1.6, 1.0, 0, 0, 0]$ with diffusivities and rotation angles reported in $\mu m^2/ms$ and radian, respectively. For each parameter, its value was fixed at a specific value within its physiologically plausible range. The remaining parameters were optimised to fit the model to the given input data. The root mean squared error (RMSE) was then reported for each parameter. A global minimum in each graph confirms that a unique solution exists for each optimisation problem.
